# Effects of Exergaming on Attentional Deficits and Dual-Tasking in Parkinson's Disease

**DOI:** 10.3389/fneur.2019.00646

**Published:** 2019-06-19

**Authors:** Eva Schaeffer, Jan-Hinrich Busch, Benjamin Roeben, Sascha Otterbein, Pavel Saraykin, Edyta Leks, Inga Liepelt-Scarfone, Matthis Synofzik, Morad Elshehabi, Walter Maetzler, Clint Hansen, Sarah Andris, Daniela Berg

**Affiliations:** ^1^Department of Neurology, Christian-Albrecht-University Kiel, Kiel, Germany; ^2^Department of Neurodegeneration, Hertie Institute for Clinical Brain Research, University of Tübingen, Tübingen, Germany; ^3^Department of Biomedical Magnetic Resonance, University of Tüebingen, Tüebingen, Germany; ^4^German Center of Neurodegenerative Diseases (DZNE), Tübingen, Germany; ^5^Mathematical Image Analysis Group, Faculty of Mathematics and Computer Science, Saarland University, Saarbrücken, Germany

**Keywords:** Parkinson's disease, exergaming, dual-tasking, cognition, quantitative motor assessment, attention

## Abstract

**Introduction:** Impairment of dual-tasking, as an attention-based primary cognitive dysfunction, is frequently observed in Parkinson's Disease (PD). The Training-PD study investigated the efficiency of exergaming, as a novel cognitive-motor training approach, to improve attention-based deficits and dual-tasking in PD when compared to healthy controls.

**Methods:** Eighteen PD patients and 17 matched healthy controls received a 6-week home-based training period of exergaming. Treatment effects were monitored using quantitative motor assessment of gait and cognitive testing as baseline and after 6 weeks of training.

**Results:** At baseline PD patients showed a significantly worse performance in several quantitative motor assessment parameters and in two items of cognitive testing. After 6 weeks of exergames training, the comparison of normal gait vs. dual-tasking in general showed an improvement of stride length in the PD group, without a gait-condition specific improvement. In the direct comparison of three different gait conditions (normal gait vs. dual-tasking calculating while walking vs. dual-tasking crossing while walking) PD patients showed a significant improvement of stride length under the dual-tasking calculating condition. This corresponded to a significant improvement in one parameter of the D2 attention test.

**Conclusions:** We conclude, that exergaming, as an easy to apply, safe technique, can improve deficits in cognitive-motor dual-tasking and attention in PD.

## Introduction

Parkinson's disease (PD) is a neurodegenerative disorder defined by its cardinal motor symptoms rigor, tremor, and bradykinesia. However, motor impairment in PD is not limited to these symptoms but includes more complex deficits of motor control and coordination. Additionally, a wide range of non-motor symptoms, including cognitive deficits, may occur, having a considerable effect on the patients' quality of life (1). Among cognitive dysfunctions, impairment of dual-tasking, as a primary cognitive deficit with a direct impact on motor and especially gait functions (2–5), is frequently observed in PD. Functional MRI studies revealed a close relation of altered attentional networks and dual-task performance (6, 7). Impaired dual-tasking can have a considerable influence on daily activities of PD patients and has been associated with an increased risk of falls (8). As there is a limited effect of pharmacological therapy or deep brain stimulation on dual-tasking (9), other strategies are sought to positively influence these deficits. Previous studies showed that both, attentional cognitive performance and dual-tasking are responsive to training interventions and have a reciprocal impact on each other (10–12).

While specific non-pharmacological interventions like physiotherapy or occupational therapy are well-known and recommended for PD, new training techniques, which deploy different motivational incentives, are increasingly applied. Another promising approach is “exergaming,” a combination of physical exercise and gaming, where patients have to control a videogame with their movements using different forms of optical or tactile sensors. Exergaming has some considerable advantages, e.g., the possibility of home-based utilization. The direct feedback on task performance as well as the highly motivational and challenging character of the games implement important aspects of cognitive engagement (13). Moreover, the simultaneous training of cognitive and motor aspects in constantly changing virtual environments is particularly suited to address dual-tasking as required for the constantly changing situations of everyday life. Exergames have been seen to improve motor and cognitive functions in neurological diseases including ataxia and stroke (14–16) and an increasing number of studies have shown promising results in PD (17–22).

Using a multidisciplinary approach, the Training-PD study set out to evaluate the effects of exergaming and other cognitive and physical training forms on neuronal plasticity, motor and non-motor function in PD in a randomized, parallel group trial. We here present data from the Training-PD exergaming part of the study on attentional motor and cognitive functions after a 6-week training intervention.

## Methods

### Subjects

In total, 64 patients were recruited between 07/2015 and 12/2017 from the outpatient clinic of the department of Neurodegeneration at the University of Tuebingen. Inclusion criteria for PD patients were: (1) diagnosis of PD according to the UK brain bank criteria and (2) Hoehn and Yahr score ≤ 2.5 (in order to enable an unguarded training at home and avoid potential injuries resulting from postural instability). After study inclusion, PD patients were randomly assigned to one of three interventions: (I) PD physiotherapy, (II) PD braingames, and (III) PD exergames. To obtain comparable groups a stratified randomization protocol was used, including age, sex, and PD disease duration. Moreover, 20 healthy controls were recruited using public notices. The Control group (IV) received the same exergaming training and was matched with regard to sex and age.

Due to logistic reasons, all interventions were performed in parallel with a priori planned separate data analyses. We here present data on exergaming training from group III (PD exergames, *n* = 18) and group IV (Controls exergames, *n* = 17).

Exclusion criteria for all study participants comprised: (1) Presence of major depression (Beck Depression Inventory >18 points); (2) physical status or diseases (other than PD) affecting physical training; (3) signs of dementia (Montreal cognitive assessment, MoCA <21); (4) Hoehn and Yahr ≥ 3 or other signs in neurological examination indicating a higher risk for falls, (5) contraindications for the performance of MRI (exploratory outcome of the study, data reported separately), and (6) planned change in medication or the usual training. Participants with mild cognitive impairment (MOCA 21-25) were not excluded from the study (23). The study protocol was approved by the local ethical committee. All subjects gave written informed consent in accordance with the Declaration of Helsinki.

### Intervention

All study participants received a training protocol for 6 weeks with three 45-min- sessions per week. Implementation of the training was documented using a training diary kept by the participants. PD patients and healthy controls received the same training protocol, using a commercially available Microsoft Kinect system (Microsoft Corporation, Redmond, WA, USA). An electronic PubMed search to identify game characteristics best suitable for PD patients regarding design and content was performed. Based on this research games focusing on two main aspects were selected: (1) games suitable to train bradykinesia, hypokinesia, and dual-tasking and (2) games using a clear design, without overwhelming visual or optic input (19, 24). Three games were chosen from the game pack “Your shape: Fitness evolved:”

- “Virtual smash” (15 min per session): participants had to shatter virtual boxes in different distances with long and fast arm swings. Fast speed and correct arm coordination resulted in higher point scores.
- “Light race” (15 min per session): participants had to step on virtual enlightened fields with long and fast steps. Fast speed and correct leg coordination resulted in higher point scores.
- “Kardio boxing” (15 min per session): participants had to follow the instructions of a virtual trainer, showing a complex, rhythmic coordination training including both, arms and legs. Correct motion sequences resulted in higher point scores.

The intensity and level of difficulty of the games adapted automatically to the participant's performance level.

At baseline each participant was individually instructed on how to use the software in a personal introductory session. Proficiency criteria were (a) the correct navigation through the program and (b) the correct performance of the games. Afterwards the training was performed at home.

### Assessments

All participants underwent quantitative motor assessment and cognitive testing before and after the training.

#### Quantitative Motor Assessment

Motor performance was quantified using the Mobility Lab® system for an objective measurement of standardized motor tasks using six body sensors (OPAL APDM, Inc., Portland, OR, United States), which had been validated earlier in elderly individuals and PD (25, 26). The system included six sensors, one at the chest, one at each wrist and ankle, and one lower back sensor. Data of four different motor tasks were used for the analyses.

##### Instrumented Timed-up-and-go (iTUG)

Participants were asked to stand up from a chair, walk 7 m in a habitual speed, turn around 180° at a specific mark, walk back, and sit down again. Total duration of the task, as well as duration and peak velocity of two specific motor aspects (sit-to-stand and turn-to-sit) were measured.

##### Instrumented Walk (iWalk) Normal Pace

Participants walked 20 m in a 3 m wide hallway in their self-selected speed.

##### iWalk Calculating

Participants were asked to walk the same route again, while solving a serial subtraction equation (minus 7) as a dual-tasking challenge. The standardized starting number for the serial subtraction was different for baseline and follow-up.

##### iWalk Crossing

Participants were asked to hold a clipboard in their non-dominant hand while walking the same distance and place crosses on a prepared document with their dominant hand.

Stride length and velocity, cadence (steps per minute), cycle time (time used for one complete gait cycle), and arm swing (velocity and range of motion, RoM) were calculated from the latter three tasks by the software's validated algorithms.

#### Cognitive Testing

All participants received the same cognitive testing including the Montreal Cognitive Assessment (MOCA), the D2 Attention test (27), the California Verbal Learning Test (CVLT, German version) (28), and the Regensburger Word Fluency test (RWT) (29).

The *MOCA* total score was used for a global overview of the participant's cognitive status. Two parameters of the *D2 Test* were used to assess the ability of the participant to concentrate on a certain task (crossing out the latter “d”): KL = amount of correctly crossed symbols minus amount of omissions and F% = percentage of mistakes in relation to edited signs. Immediate, short and long delay free recall of the *CVLT* were used to evaluate episodic memory. Four subtests of the *RWT* measured word fluency and executive function. Percentage ranks of the *D2* and *RWT* were corrected for gender, age and education.

To rule out learning effects parallel-test versions for the CVLT (30) and RWT (31) were used for baseline and follow-up.

### Statistics

Preprocessing of the Mobility Lab® data was performed using Matlab (Version R2016b, The Mathworks Inc., 1984). SPSS 24.0 (SPSS Inc., IBM, USA) was used for statistical analyses. Statistical distribution was tested using the Kolmogorov-Smirnov-test.

For *cross-sectional* group comparisons at baseline and follow-up, the Mann-Whitney-*U*-test was used for non-normally distributed variables and student's *t*-test for normally distributed variables. To identify parameters of the Mobility Lab® assessment indicating disease-specific deficits, in a first, exploratory analyses a group comparison of PD patients vs. healthy controls was performed at baseline. Parameters identified to be significantly different (*p* < 0.05) where used for further longitudinal analyses. Parameters of cross-sectional analyses were not corrected for multiple comparisons as the main outcome purpose of the study was the evaluation of longitudinal intervention effects.

*Response-to-intervention analyses* were performed with a two-way repeated measures ANOVA (time × group). To compare the different gait challenges of the *iWalk* task, the factor “gait condition” was added (time × group × gait condition). In a first analyses normal gait was compared to gait under dual-tasking conditions in general (iWalk: normal pace vs. iWalk calculating + iWalk crossing subsumed), with a planned nested analyses of the two dual-tasking conditions. In a second analyses all different gait conditions were compared directly (iWalk: normal pace vs. iWalk calculating vs. iWalk crossing). Non-normally distributed data were log-transformed.

Correlations were performed using the Pearson's correlation coefficient (one-tailed).

A *p* < 0.05 was accepted as statistically significant. For better readability, data are presented with mean (standard deviation).

## Results

### Group Characteristics

Two PD patients and two healthy controls reported an incomplete training or major deviations from the training protocol and were thus excluded from the analyses. Only minor aberrations of the training protocol (e.g., missing of one training session) were accepted. The total exercise time of both groups was comparable (*p* = 0.42). Mean age was 58.6 (9.9) for the PD and 57.8 (11.4) for the Control group. 55.6% of the PD group and 52.9% of the healthy control group were male. MOCA total score at baseline was comparable between PD patients (Mean: 27.3) and Controls (Mean: 27.2) (minimal score: 22). Median disease duration of PD patients was 4 years (1–20) and the mean levodopa-equivalent dosage was 340 mg per day (100–925). The median total score of part III of the Movement Disorder Society Rating Scale (MDS-UPDRS) was 28 (7–38). Seventeen PD patients were rated Hoehn & Yahr stage 2, one patient was rated Hoehn & Yahr stage 2.5.

### Quantitative Motor Assessment

#### Between-Group Comparisons at Baseline and at Follow-Up

At baseline 13 parameters of the Mobility Lab® were significantly different between PD patients and Controls and were therefore used for further group comparisons. PD patients were significantly slower in the following parameters of the iTUG test: total duration, Sit-to-stand peak velocity, Turn-to-sit duration, and peak velocity. Moreover, they showed significant lower values in the following parameters: stride length and stride velocity in all iWalk tasks, peak arm swing velocity in iWalk normal pace, and dual-tasking calculating and arm swing range of motion in iWalk dual-tasking calculating. No significant differences were observed in cadence and cycle time of all iWalk assessments and for the duration from sitting to standing during the iTUG task. At follow-up two parameters showed no significant group difference between PD and Controls anymore: stride length and range of motion under dual-tasking calculating conditions (Table 1).

**Table 1:** Quantitative motor assessment—between-group comparisons at baseline and at follow-up.

	**PD *n* = 18**	**Controls *n* = 17**	***p*-value**
**STRIDE LENGTH (%STATURE)**			
**iWALK: normal pace**			
– Baseline	83.1 (5.4)	89.9 (5.7)	**0.002**
– Follow-up	83.6 (5.3)	89.2 (5.1)	**0.018**
**iWALK: dual-tasking—calculating**			
– Baseline	79.2 (8.8)	89.9 (5.7)	**<0.001**
– Follow-up	85.1 (6.4)	90.0 (4.8)	0.05
**iWALK: dual-tasking—crossing**			
– Baseline	79.0 (8.2)	87.0 (5.9)	**0.004**
– Follow-up	79.6 (8.4)	87.4 (4.8)	**0.021**
**STRIDE VELOCITY (%STATURE/s)**			
**iWALK: normal pace**			
– Baseline	79.6 (6.8)	86.9 (7.7)	**0.009**
– Follow-up	79.0 (7.8)	87.0 (9.0)	**0.012**
**iWALK: dual-tasking—calculating**			
– Baseline	76.0 (11.4)	90.3 (7.9)	**<0.001**
– Follow-up	88.7 (10.6)	98.4 (7.0)	**0.006**
**iWALK: dual-tasking—crossing**			
– Baseline	77.4 (9.6)	89.5 (11.7)	**0.004**
– Follow-up	78.6 (12.0)	90.3 (9.6)	**0.005**
**PEAK ARM SWING VELOCITY (°/s)**			
**iWalk: normal pace**			
– Baseline	173.4 (61.3)	221.3 (73.6)	**0.040**
– Follow-up	172.4 (82.2)	237.7 (48.1)	**0.004**
**iWalk: dual-tasking calculating**			
– Baseline	188.9 (88.1)	275.0 (102.5)	0.015
– Follow-up	228.8 (90.4)	316.7 (74.1)	0.009
**RoM ARM (°)**			
**iWALK: dual-tasking—calculating**			
– Baseline	20.4 (15.9)	35.3 (15.6)	**0.007**
– Follow-up	26.4 (16.4)	34.8 (14.2)	0.09

*Values are given as mean and standard deviation; iWALK, instrumented walk; n, number; p, level of significance; PD, Parkinson's Disease; RoM, Range of motion; s, seconds. Bold values represent significant values*.

#### Response-to-Intervention Analyses

Both groups required significantly less time for the iTUG test after 6 weeks of exergaming (*p* = 0.005), without significant group differences. In the comparison of stride length under normal vs. dual-tasking conditions (subsumed calculating and crossing), a significant group effect was seen in favor of the PD group. No significant differences were seen for the time × gait condition and time × group × gait condition analysis. Therefore the additional nested analysis of the dual-tasking conditions crossing vs. calculating was not applicable. When comparing all three gait conditions directly a significant improvement of stride length with a significant interaction of time × group × gait condition was seen in favor of the PD group under dual-tasking calculating conditions (Table 2). Peak arm swing velocity improved in both groups (*p* = 0.024) without significant group or gait condition effects. No significant improvement was seen for stride velocity and range of motion of the arm.

**Table 2:** Quantitative motor assessment—response-to-intervention analyses in PD vs. controls.

***iWalk: Stride length*** **PD (*****n*** **=** **18) vs. Controls (*****n*** **=** **17)**			
***p*****-value** **time**	***p*****-value** **time** **×** **group**	***p*****-value** **time** **×** **gait condition**	***p*****-value** **time** **×** **group** **×** **gait condition**
**iWalk: normal pace vs. iWalk: dual-tasking (calculating** **+** **crossing)**			
0.09	**0.040**	0.07	0.36
**iWalk: normal pace vs. iWalk: dual-tasking—calculating** vs. **iWalk: dual-tasking—crossing**			
**0.008**	**0.009**	**0.014**	0.023^ϕ^^‡^^*^

*iWALK, instrumented walk; n, number; p, level of significance; PD, Parkinson's Disease. time: change from baseline to follow-up; group: PD vs. Controls; gait condition: normal pace vs. dual-tasking calculating vs. dual-tasking crosses ^ϕ^normal pace vs. dual-tasking calculating: p = 0.036*;

‡ *normal pace vs. dual-tasking crosses: p = 0.64*;

* *dual-tasking calculating vs. dual-tasking crosses: p = 0.018. Bold values represent significant values*.

### Cognition

#### Between-Group Comparisons at Baseline

At baseline, PD patients scored significantly worse than healthy controls in one item of the D2 Attention test (KL = amount of correctly crossed symbols minus amount of omissions) and in one item of the RWT (category change lexical) (Table 3).

**Table 3:** Cognition—Response-to-intervention analyses in PD vs. Controls.

	**PD *n* = 18**	**Controls *n* = 17**	***p*-value group**	***p*-value time**	*p*-value time * group
**D2 TEST OF ATTENTION**					
– **F% (PR)**					
– Baseline	58.7 (27.7)	55.9 (19.1)	0.56	0.20	0.29
– Follow-up	68.8 (22.3)	58.9 (19.6)	0.11		
– **KL (PR)**					
– Baseline	27.1 (23.1)	56.1 (26.2)	**0.002**	**0.029**	**0.015**
– Follow-up	40.7 (23.2)	61.1 (33.5)	**0.042**		
***CVLT***					
– **Immediate recall (PR)**					
– Baseline	54.1 (12.1)	60.7 (11.0)	0.11	0.62	0.37
– Follow-up	56.2 (9.3)	58.7 (11.8)	0.50		
– **Short delay free recall (PR)**					
– Baseline	11.4 (3.2)	12.3 (2.7)	0.51	0.78	0.09
– Follow-up	12.2 (2.5)	11.4 (3.5)	0.70		
– **Long delay free recall (PR)**					
– Baseline	12.1 (2.4)	11.6 (2.7)	0.46	0.45	0.65
– Follow-up	12.5 (3.1)	11.8 (3.3)	0.70		
***RWT***					
– **Word fluency: lexical (2 min)**					
– Baseline	56.6 (28.4)	46.6 (24.3)	0.35	0.84	0.51
– Follow-up	58.6 (30.5)	51.7 (25.1)	0.33		
– **Category change: lexical (2 min)**					
– Baseline	40.7 (27.9)	59.4 (22.4)	**0.042**	0.58	0.24
– Follow-up	52.4 (29.9)	56.9 (28.0)	0.62		
– **Word fluency: semantic (2 min)**					
– Baseline	45.6 (27.2)	47.8 (25.9)	0.83	**0.040**	0.37
– Follow-up	53.0 (25.7)	56.1 (28.0)	0.57		
– **Category change: semantic (2 min)**					
– Baseline	45.9 (30.3)	52.7 (22.5)	0.47	0.34	0.58
– Follow-up	47.9 (31.6)	60.0 (25.8)	0.23		

*Values are given as mean and standard deviation; CVLT, California Verbal Learning Test; F%, Percentage of mistakes in relation to edited signs; KL, Total of correct edited signs minus total of wrong edited signs; min, minutes; MOCA, Montreal Cognitive Assessment; n, number; p, level of significance; PD, Parkinson's Disease; PR, percentage range; RWT, Regensburger word fluency test. Bold values represent significant values*.

#### Response-to-Intervention Analyses

Both, PD patients and Controls showed a significant increase of semantic word fluency and an improved cognitive performance in the D2 test of attention (KL) after the intervention. Improvement of the D2 test was significantly more pronounced in PD patients than in Controls (Table 3).

## Correlations

The improvement of stride length under cognitive-motor dual-tasking conditions (iWalk: dual-tasking—calculating, V2-V1) correlated significantly with the improvement of the D2 test of attention (KL, V2-V1) (*p*-level 0.05).

## Discussion

Our results show a significant improvement of deficits in motor and cognitive performance of attention-based tasks after 6 weeks of exergaming training in PD patients.

Quantitative motor assessment revealed a significant improvement on a single parameter level, i.e., stride length in PD patients under dual-tasking conditions (walking while calculating) after exergames training when compared to healthy controls. This is of particular interest, as a dysregulation in stride length has been identified as a key element for gait impairment in PD patients (32), correlating with disease progression (33). The targeted improvement of this deficit is therefore of high clinical relevance for PD patients. Interestingly, our results could not reveal an improvement of the “motor-motor” dual-tasking condition (crossing while walking), which was also reflected by a non-significant result when comparing normal gait vs. gait under dual-tasking conditions in general (with the calculating and crossing task subsumed). These results suggest that exergaming does not improve dual-tasking in general in PD patients, but only certain aspects, which is in line with previous studies (34, 35). We therefore hypothesize that the effect observed on calculating, but not crossing while walking, might reflect a specific improvement of cognitive-motor but not motor-motor dual-tasking.

Additionally, these results correspond well with the cognitive performance. PD patients showed a significantly worse performance than healthy controls in concentration capacity as measured by the D2 test at BL, in line with previous studies, identifying attentional deficits in PD as an important component of cognitive impairment in all stages of PD (36, 37). However, a significant, group-dependent improvement of concentration capacity in PD patients was observed after exergames. Taken together, the corresponding improvement in concentration capacity and a primary cognitive dual-task might reflect a targeted improvement of attention-based tasks with high everyday relevance.

Besides the improvement of attention-based tasks, one iTUG variable measuring motor performance (total duration) improved in both groups, without significant group differences. We conclude that this observed improvement reflects a learning of the motion sequence, independently from pre-existing functional deficits.

Several limitations of the study have to be addressed, including first the limited number of participants. Second, it must be underlined that the two conditions walking while calculating and walking while crossing are both subtypes of dual-tasking. Therefore the analyses of normal gait vs. dual-tasking gait in general (with a nested factor calculating vs. crossing) would have been the preferred statistical method, but showed no significant differences in our study. However, previous studies showed that the complexity of the simultaneously performed task has a major impact on walking performance (38). Similarly, our data suggest that a mixed cognitive-motor dual-tasking challenge might require attentional capacity in a different way than a “motor-motor” dual-task. We therefore conclude that it is justified to analyze these gait conditions separately. Additionally, the use of home-based training documented by patient diaries lacks a strict external control of the performed training intensity, which may result in shortening of the exact training protocol in this study. However, the current study placed emphasis on feasibility for the patients and future exergame systems are likely to record information on training intensity as well. Finally, it must be discussed whether the improvement of motor and cognitive performance was due to re-test learning effects at follow-up. However, the correlation of an improvement in both attentional motor and cognitive tasks specifically in the PD group, suggests rather a specific intervention-driven and disease-dependent effect. Moreover, the randomization of two versions for the dual-tasking calculating task reduces the risk of simple learning effects. Moreover, the study did not include a PD control group without a training intervention, therefore placebo effects of the intervention cannot be ruled out.

Taken together, results of the Training-PD study indicate that specific aspects of dual-tasking, as a complex interaction of motor and cognitive function, can be improved by exergaming. These findings go in line with previous studies showing an improvement of gait parameters or cognitive function after specific motor-cognitive dual-tasking training (39–44).

Especially considering the very limited effect of pharmacological treatment (45), the high correlation with an increased risk of falls and impaired quality of life (46–49), the possibility to improve attentional deficits through exercise is of high clinical relevance for PD patients. Exergaming, combining feedback mechanisms, motivation and simultaneous motor-cognitive activation, might be particularly well-suited to address attentional deficits. It can therefore be considered a suitable alternative or add-on to the gold-standard physiotherapy to improve dual-tasking in PD (50, 51). Our results should be confirmed in future studies that assess long-term-effects of exergaming and may also investigate underlying (patho)mechanisms of different dual-tasking conditions.

## Data Availability

The datasets generated for this study are available on request to the corresponding author.

## Ethics Statement

This study was carried out in accordance with the recommendations of the ethical committee of the Eberhard-Karls-University of Tuebingen, with written informed consent from all subjects. All subjects gave written informed consent in accordance with the Declaration of Helsinki. The protocol was approved by the ethical committee of the Eberhard-Karls-University of Tuebingen.

## Author Contributions

ES, IL-S, MS, WM, and DB were responsible for conception, design, and organization of the study. ES, J-HB, BR, SO, PS, EL, IL-S, ME, and SA were responsible for execution of the study. ES, ME, and CH performed the statistical analysis. ES wrote the manuscript. All authors have contributed significantly and carefully reviewed this manuscript.

### Conflict of Interest Statement

ES reports speaker's honoraria from Bayer Vital GmbH and Novartis outside the submitted work. BR has received financial support for research training at the University of Oxford within the CENTRE-PD project funded by the European Union's Horizon 2020 research and innovation programme (grant agreement No 692320) and reports personal fees from Actelion Pharmaceuticals Ltd., outside the submitted work. IL-S reports grants from AKF Grant (Institutional), during the conduct of the study; grants from Johnson & Johnson, grants from European Commission, H2020-TWINN-2015, grants from “Parkinson National Center of Excellence in Research (NCER-PD),” grants from Michael J. Fox Foundation, outside the submitted work. MS reports grants and personal fees from Actelion Pharmaceuticals, outside the submitted work. WM reports grants and personal fees from Lundbeck, grants from Neuroalliance, grants from Janssen, grants from UCB, personal fees from Rölke, personal fees from Abbvie, outside the submitted work; in addition, WM has a patent for the assessment of dyskinesias licensed to German patent office, 102015220741.2. DB reports grants from Janssen Pharmaceutica, grants from Michael J. Fox Foundation, grants from Damp foundation, grants from German Parkinson's Disease Association (dPV), grants from BMWi, grants from BMBF, grants from Parkinson Fonds Deutschland GmbH, grants and speaker's honoraria from and consultancies for UCB Pharma GmbH, grants and speaker's honoraria from TEVA Pharma GmbH, grants from and consultancies for Novartis Pharma GmbH, grants and speaker's honoraria from and consultancies for Lundbeck speaker's honoraria from and consultancies for BIAL, speaker's honoraria from and consultancies for Biogen, honoraria from Bayer, outside the submitted work. The remaining authors declare that the research was conducted in the absence of any commercial or financial relationships that could be construed as a potential conflict of interest.
